# Regional coordination in medical emergencies and major incidents; plan, execute and teach

**DOI:** 10.1186/1757-7241-17-32

**Published:** 2009-07-20

**Authors:** Amir Khorram-Manesh, Annika Hedelin, Per Örtenwall

**Affiliations:** 1 Prehospital and Disaster Medicine Centre, Gothenburg, Sweden

## Abstract

**Background:**

Although disasters and major incidents are difficult to predict, the results can be mitigated through planning, training and coordinated management of available resources. Following a fire in a disco in Gothenburg, causing 63 deaths and over 200 casualties, a medical disaster response centre was created. The center was given the task to coordinate risk assessments, disaster planning and training of staff within the region and on an executive level, to be the point of contact (POC) with authority to act as "gold control," *i.e*. to take immediate strategic command over all medical resources within the region if needed. The aim of this study was to find out if the centre had achieved its tasks by analyzing its activities.

**Methods:**

All details concerning alerts of the regional POC was entered a web-based log by the duty officer. The data registered in this database was analyzed during a 3-year period.

**Results:**

There was an increase in number of alerts between 2006 and 2008, which resulted in 6293 activities including risk assessments and 4473 contacts with major institutions or key persons to coordinate or initiate actions. Eighty five percent of the missions were completed within 24 h. Twenty eight exercises were performed of which 4 lasted more than 24 h. The centre also offered 145 courses in disaster and emergency medicine and crisis communication.

**Conclusion:**

The data presented in this study indicates that the center had achieved its primary tasks. Such regional organization with executive, planning, teaching and training responsibilities offers possibilities for planning, teaching and training disaster medicine by giving immediate feed-back based on real incidents.

## Background

### Introduction

To be able to cope with the implications, both quantitative and qualitative, of a disaster, basic healthcare infrastructure needs to be expanded and adapted [1-3]. The involved organizations need to be coordinated and follow pre-defined response plans, command and control systems and support functions to counter the substantial challenges presented at the scenes [4-6]. Region Västra Götaland in Sweden, formed in 1999 by merging 4 previous County Councils, has responded to this by the creating a center that has the formal position to be contacted about potential major incidents/disasters, to act as a crisis management center and to provide training in disaster medicine. The region, roughly a triangle with 300 km sides, is a prominent industrial zone in Sweden with 1.5 million inhabitants (17% of the overall Swedish population), living in urban as well as rural, and scarcely populated areas. Scandinavia's largest port in Gothenburg, automotive factories, refineries, chemical and pyrotechnical industries, several airports, major highways, shipping and public gatherings all need to be included in the risk assessment regarding possible major incidents in this region. The purpose of this study was to find whether this institution has achieved its primary tasks by analyzing its registry during January 1^st ^2006 until December 31^st ^2008.

### Setting

**Figure 1:**
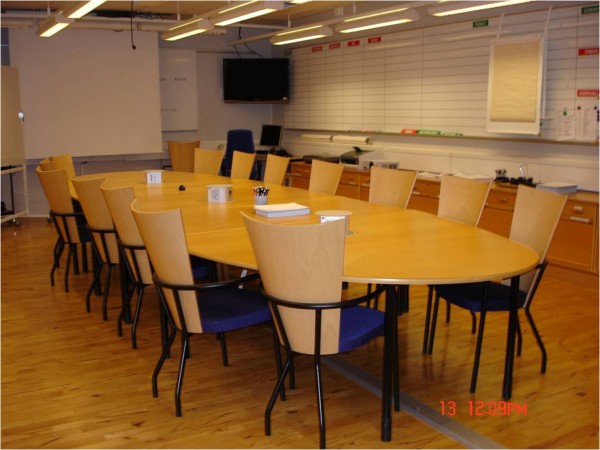
**Shows the gold command and control room**.

**Figure 2:**
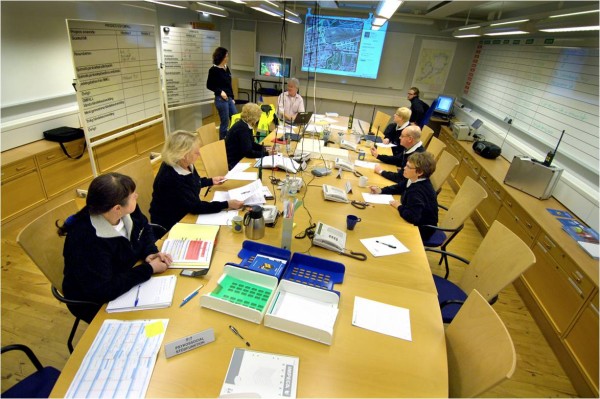
**Gold command and control centre in action**.

According to Swedish law, the healthcare services are responsible for offering emergency medical care to the public. In Region Västra Götaland this service is provided through 150 primary healthcare centers, 10 emergency hospitals and a hospital integrated EMS (including HEMS) [7,8]. Region Västra Götaland has seen numerous major incidents. In 1998 a fire in a disco in Gothenburg caused 63 fatalities and more than 200 casualties, most of them teenagers. The following investigation revealed certain short-comings regarding the medical response, recognizing the need of a regional point of contact ("POC") and command and control centre for the health care services. In 1999 PKMC (Prehospital Disaster Medicine Centre) was established with the tasks to plan for, train for, and immediately assume regional command and control in case of major incidents involving the healthcare sector [7,9]. The centre's premises were made suitable for running command and control over days and weeks with secure communications, back-up generators for power, white boards, computers, etc. The staff was trained to handle all support functions within the command and control centre (Figure 1 and 2).

A system with a duty officer (RTiB) (RN, specialized in emergency care combined with further training in disaster medicine as well as in depth knowledge about the available regional medical resources) and a back-up physician on call on weekly (RBL; a senior surgeon or anesthesiologist with training in disaster medicine) was created. In this 24/7 system, the RTiB is the POC for the healthcare facilities within the region and has the mandate to act as "Gold Control," *i.e*. to take immediate strategic command over all regional medical resources [7]. Most alerts (> 90 %) are handled by RTiB (4 persons). However they may mediate and inform other authorities to initiate actions.

The EMS dispatch centre (SOS Alarm) is instructed to page the RTiB on certain criteria (Appendix 1). The RTiB is requested to respond within 5 min after being paged. If needed the RTiB may page RBL, who normally works at one of the hospitals within the region and is requested to respond within 15 min. The other employees at PKMC (7 staff) were in cases of major incidents assigned to work as staff members at the Regional command and control centre established within the centers' premises. Specialists in other fields (*e.g*. nuclear medicine, hazmat, infectious diseases) could be summoned to the centre when needed. All data is recorded in a registry and may easily be analyzed.

## Materials and methods

***Alert ***was defined as a warning signal and threat, which might result in a) an ***incident ***defined as a single distinct event or a public disturbance or to b) an ***alarm***, defined as a fear or dismay. All data concerning an alert is registered in a log. This registry (PKMC-registry) started in 1999, and was initially paper-based, but since 2006-01-01, a web-based log (Saltwater™) has been used [10]. The information is available from any computer with an Internet connection, allowing multiple users to be on-line simultaneously. Based on the nature of alerts, RTiB ***undertook ***(made an action as POC such as initiation of a disaster plan, redistributing of regional resources) or ***mediated ***(informed other authorities to take actions) an action. Activities are time-stamped as they are entered and data are mirrored on two separate servers.

Data were organized in pre-defined variables to cover a wide field of incidents. However, there are open fields to complete or add data if necessary. The data from this registry between 2006-01-01 and 2008-12-31 has been transferred to Excel (Microsoft Corp, USA) for review and analysis, presented as below. When needed the results were presented in mean ± SD.

1. Number of alerts (weekdays, months, and number of people involved)

2. Demography (regional, national, within Europe, outside Europe)

3. Type of alerts

a. Incidents

b. Alarms

4. Resulting activities

a. Undertaken

b. Mediated

5. Workload (0–4 h, 4–12 h, 12–24 h, and > 24 h)

6. Training, exercises and Education

## Results

### Number and causes of alerts

Registered alerts were 324 in 2006, 338 in 2007 and 445 in 2008. There was a 30% increase in number of alerts between 2006 and 2008 (Table 1). The number of alerts designated as "hospital-related" increased as well as terror and threats, information technology malfunctions, public and sport gatherings. "Hospital related" incidents refer to situations where the emergency hospitals, for various reasons, were not able to function with full capacity. Shortage of available beds (especially intensive care units beds), staff shortage, CT (Computed Tomography) scanner breakdown or maintenance, emergency department overcrowding were some of the causes and the result was ambulance diversions and secondary overloading of the nearest hospital. On the contrary, the number of traffic crashes showed a slight reduction. There was no common denominator between months of the year or days of the week regarding registered alerts.

**Table 1:** Causes of alerts

	2006	2007	2008
Hospital related	4	11	61

Terror/Threat	8	10	15

Traffic crashes	180	173	164

Sea	12	1	2

Sport events	17	13	27

Police	17	27	35

Public gathering	7	8	27

Chemical and Infectious events	17	15	19

Fire/Flooding	40	33	46

International	3	4	3

National	9	7	25

Nature	4	1	11

Information/weather/Others	6	35	10

Total	324	338	445

### Demography

The number of alerts emanating from events within Gothenburg has increased steadily due to hospital-related events (in the city as well as in the region with secondary impact on the hospitals in Gothenburg). Actions concerning international incidents remained at a low level (Table 2).

**Table 2:** Number of alerts, resulted activities, contacts, location, and workload

	2006	2007	2008	Total
Alerts	324	338	445	1107

Resulted activities	2408	1577	2308	6293

Contacts, Communications	1814	1116	1543	4473

Local (within Gothenburg)	119	135	148	402

Regional	320	336	409	1065

National	4	8	31	43

European	3	0	1	4

Outside Europe	5	2	4	11

Exercises	8	8	12	28

Workload				

> 24 h	30	34	107	171

12–24 h	8	16	40	64

4–12 h	17	24	55	96

< 4 h	269	263	244	776

### Type of alerts; Incidents and alarms

There were 64 various causes of alerts, which were further grouped under 13 different headings in this study for simplicity (Table 1). For example, all traffic crashes, predefined as car accidents, truck accidents and so on were grouped in one.

### Resulting activities

Each alert resulted in one or more activities by the RTiB. Some 6293 activities were registered in response to a total of 1107 alerts (Table 2). RTiB registered 4473 contacts with major institutions or key persons. Most calls were made to the ambulance services (single ambulances/ambulance officers on duty), SOS alarm (the EMS dispatch centre), other emergency services (Police, Fire & Rescue departments), hospitals and the National Board of Health and Welfare (Table 2). In about 5–10% of cases the RBL were contacted due to the medical nature of the case and the possibility of regional or national/international involvement.

### The workload

A total number of 936 activities resulted in actions that were completed within 24 h and mostly (776) < 4 h. However, 171 missions lasted more than 24 h. Detailed information about these missions is presented in table 3. Swedish citizens' evacuation from Lebanon, in the wake of the Israeli attack in 2006, was the most time-consuming mission. This conflict resulted in continuous running of PKMC's command and control centre (24 h/day) during 21 days, involving all staff. PKMC was tasked by the National Board of Health and Welfare to send medical teams (nurses and physicians) from Region Västra Götaland to Lebanon, Cyprus and Syria as well as to coordinate all possible secondary air Medevacs of Swedish citizens brought from the area to Stockholm/Arlanda airport. Other long-lasting missions have been a visit by NATO military ships (15 days), storm with flooding (12 days), European Championship in track and field sports (10 days) as well as a bus crash (10 days). Since some of these events were focused on risk reduction and emergency response pre-planning as well as psychosocial support, the workload could mainly be handled during normal office hours.

**Table 3:** Detailed information about alerts lasted more than 24 hours (2006–2008)

	Time (h**)				
Incidents	Number	mean ± SD	R*	N*	I*
Hospital-related	45	53 ± 117	44	1	

Terror/Threat	4	184 ± 151	2		2

Traffic crashes	9	105 ± 176	7	1	1

Sea	2	10 ± 7	2		

Sport events	34	109 ± 277	34		

Police	26	79 ± 85	25		1

Public gatherings	13	54 ± 58	13		

Chemical and infectious events	12	145 ± 127	9	3	

Fire/Flooding	8	64 + 53	6		2

International	1	6766 ± 0			1

National	7	141 ± 350	7		

Nature	5	94 ± 96	5		

Information/weather/Others	5	69 ± 90	5		

Total	171		159	5	7

* R: Regional, N: National, I: International

** Shows the time it took to handle an incident (start and end of activities) and does not represent the active time.

### Training, Exercises and Education

During the period of study 28 exercises were performed of which 4 lasted more than 24 h (Table 2). The centre also offered numerous courses (n = 145) in Major Incident Medical Management and Support (MIMMS™) and other related courses in association with Advanced Life Support Group [11]. A continuous yearly program for updating all RTiB and RBL was running during these 3 years. The centre also offered yearly courses in command and control in cooperation with other authorities to discuss and coordinate the line of action during a disaster [7].

## Discussion

There is a need for adaptation and expansion of basic healthcare infrastructure to cope with all implications of a disaster. Such transformation may be possible through research, education and exercises. In the current study, we report how Region Västra Götaland in Sweden has created a center with the formal position to act as POC for potential disasters, to act as a crisis management center for the healthcare services and also to provide training in disaster management.

An effective disaster response depends on structured and organized cooperation and communication between different agencies/services, institutions and individuals [3]. The lack of, or deficiencies in understanding, coordination, communication and a jointly trained organization have been recognized as important factors in failure to respond properly to disasters and major incidents [3,12]. A very clear governing body is desirable to further improve the delivery of aid and to maximize resources [3,5,12]. Studies within the field of trauma care have shown that experience, training and strict protocols are important factors to improve the outcome. Therefore, regional medical operation centers have been established in many countries to tune up disaster response and reduce mortality [3,13-16].

Data from this registry showed an increase in the number of alerts, which might be due to earlier activation of RTiB by SOS Alarm on a relatively low suspicion of an emerging major incident (Appendix 1). It might also reflect the global awareness of disasters and terror-related incidents in the aftermath of disasters such as the 9/11 and the South-East Asian Tsunami when a psychological fearfulness for replication in a new time and zone exists [4-6]. Thus, often the anticipation of some major incidents necessitated performance of risk management by the centre's staff. Although the number of alerts was rather stable, the duration and intensity of consequent activities varied. The data concerning the increase in mass-gatherings and sport events in the region are vital for planning and distributing the regional resources. The high number of measures and contacts taken during these activities demonstrate the absolute need for communication and coordination (Table 2). To assert perfect and desirable ground for communication and coordination with other agencies *e.g*. Police, Fire and Rescue departments and EMS, the centre organizes continuous dialog meetings. These authorities are also invited to send staff as participants in the centre's various courses in disaster and disaster-related subjects. Personal knowledge about other agencies and their staff, gained during these activities, seems to be one of the most valuable factors in enhancing collaboration, when real major incident strikes.

During the study period, the number of local incidents decreased in favor of national and international incidents, which is a simple indicator of the globalization of the world [8,15]. It also emphasizes the permanent need for international cooperation based on common language and education; one of the main reasons for PKMC's cooperation with ALSG, UK [11]. Similar centers with redundant power to coordinate and communicate during a disaster have been reported in the literature [3,17]. However, to the best of our knowledge few, if any, have the regional responsibility for staff training by conducting disaster and disaster-related courses and training. The involvement of the same people in both planning for emergencies and disasters, training the staff for such events as well as executing the emergency and disaster plans in real life, adds strength to the organization. No shorter feed-back loop between planning and executing can exist!

The increased number of hospital-related alerts during the study period raises concern, since it has a negative impact on preparedness ("surge capacity") for medical emergencies as well as major incidents within the affected area. This has been reported by other investigators [17-19], but seems to be a new and emerging problem for Sweden. The reduction of hospital beds as a consequence of economic constraint, increased sub-specialization of hospitals as well as increased dependency on high-tech equipments can be factors contributing to this problem, making the whole healthcare system more vulnerable in case of major incidents [20].

There are some limitations imposed to our study by its retrospective design and lack of primary relevant research questions. In addition the database was not primarily designed for research, thus, there is lack of clear definitions and operating rules for the data set. However, this registry is the tool, which for the first time has recorded these events. Although this is a retrospective study, the use of a web-based system reduces some of the limitation a retrospective study may have, *e.g*. standardization of data input, and open up for new studies such as evaluation of ambulance transport (diversion and secondary transports) or evaluation of hospital bed resources; information needed for politicians to make important healthcare and socio-economical decisions. These data may also emphasize the importance of research and education within the field of disaster medicine.

In conclusion, disasters are inevitable, but can be mitigated through data accumulation, planning, educating, research and practice. To coordinate these tasks regional centers with redundant authorizations are needed. The combination of risk assessment, disaster planning and training of staff together with executive responsibility at the time of disaster may not only reveal various short-comings within our organizations and the healthcare system, but may also prevent the disastrous outcome and consequences of such short-comings.

## Appendix

### Appendix 1: Alarm criteria

1. three or more ambulances dispatched to a single incident

2. more than one hospital is expected to be involved

3. potential threat which may cause multiple casualties

4. other authorities/emergency services request contact

## Competing interests

The authors declare that they have no competing interests.

## Authors' contributions

AK conceived and designed the study. AK, AH and PÖ performed the data analysis. AK drafted the manuscript. All authors interpreted data and critically revised the manuscript. All authors have read and approved the final manuscript
